# Post-Translational Modification of Cysteines: A Key Determinant of Endoplasmic Reticulum-Mitochondria Contacts (MERCs)

**DOI:** 10.1177/25152564211001213

**Published:** 2021-03-24

**Authors:** Arthur Bassot, Junsheng Chen, Thomas Simmen

**Affiliations:** Department of Cell Biology, Faculty of Medicine and Dentistry, University of Alberta, Edmonton, Alberta, Canada

**Keywords:** endoplasmic reticulum, mitochondria, redox, cysteines

## Abstract

Cells must adjust their redox state to an ever-changing environment that could otherwise result in compromised homeostasis. An obvious way to adapt to changing redox conditions depends on cysteine post-translational modifications (PTMs) to adapt conformation, localization, interactions and catalytic activation of proteins. Such PTMs should occur preferentially in the proximity of oxidative stress sources. A particular concentration of these sources is found near membranes where the endoplasmic reticulum (ER) and the mitochondria interact on domains called MERCs (Mitochondria-Endoplasmic Reticulum Contacts). Here, fine inter-organelle communication controls metabolic homeostasis. MERCs achieve this goal through fluxes of Ca^2+^ ions and inter-organellar lipid exchange. Reactive oxygen species (ROS) that cause PTMs of mitochondria-associated membrane (MAM) proteins determine these intertwined MERC functions. Chronic changes of the pattern of these PTMs not only control physiological processes such as the circadian clock but could also lead to or worsen many human disorders such as cancer and neurodegenerative diseases.

## Introduction

Interactions between mitochondria and the Endoplasmic Reticulum (ER) were discovered in 1952 using electron microscopy of rat liver, where contacts between these two organelles depend on the nutritional status of the animal (Bernhard et al., 1952). This insight suggests that material exchange could occur in a controlled manner on these contacts, a hypothesis confirmed and characterized with the discovery of "Mitochondria-associated ER membranes" (MAMs) as a lipid transfer platform (Vance, 1990) and a site of Ca^2+^ flux (Rizzuto et al., 1998). Accordingly, the biochemical MAM isolate contains enzymes necessary for phospholipid synthesis on either side of the mitochondria-ER contact (MERC) structure (Vance, 1991), as well as Ca^2+^ channels and pumps on the ER and mitochondria that maintain a circular Ca^2+^ equilibrium (Raffaello et al., 2016). Within the ER, this equilibrium determines oxidative protein folding through Ca^2+^-dependent chaperones (Margittai and Sitia, 2011; Braakman and Hebert, 2013), while within mitochondria, it controls energy production and apoptosis through Krebs cycle dehydrogenases and the permeability transition pore, respectively (Bauer and Murphy, 2020). Moreover, MERCs fulfill structural roles in mitochondrial fusion and fission mechanisms (Friedman et al., 2011), in autophagosome formation (Hamasaki et al., 2013) and as a lipid synthesis hub that may eventually foster lipid droplets (Vance, 2014). Consistent with this array of functions, the MERC proteome is now recognized to comprise ER Ca^2+^ release channels (e.g., inositol 1,4,5 trisphosphate receptor type 3, IP_3_R3), ER Ca^2+^ uptake pumps, ER protein folding enzymes (e.g., calnexin), mitochondrial Ca^2+^ handling proteins (e.g., voltage dependent anion channel 1, VDAC1), mitochondrial fission and fusion mediators (e.g., dynamin-related protein 1, Drp1, mitofusin-2), lipid metabolizing enzymes (e.g., acetyl-CoA acetyltransferase, ACAT1) and proteins involved in autophagosome formation (e.g., Rab32) (Ilacqua et al., 2017). The MERC functions and its proteome determine how closely the respective membranes approach each other within a distance range of 0 to 100 nm (Giacomello and Pellegrini, 2016).

MERC-localized proteins can undergo posttranslational modifications (PTMs) such as phosphorylation or palmitoylation, which have functional implications. For instance, calnexin phosphorylation dependent on protein kinase CK2 and ERK determines its interaction with phosphofurin acidic cluster sorting protein 2 (PACS-2), a critical regulator of MERC formation (Simmen et al., 2005) that controls the extent of calnexin MAM enrichment (Myhill et al., 2008). Similarly, calnexin palmitoylation also promotes its enrichment to MAMs (Lynes et al., 2012). Recent research has identified redox-controlled PTMs on cysteines as a novel key determinant of MERC function and formation.

**Figure 1.**
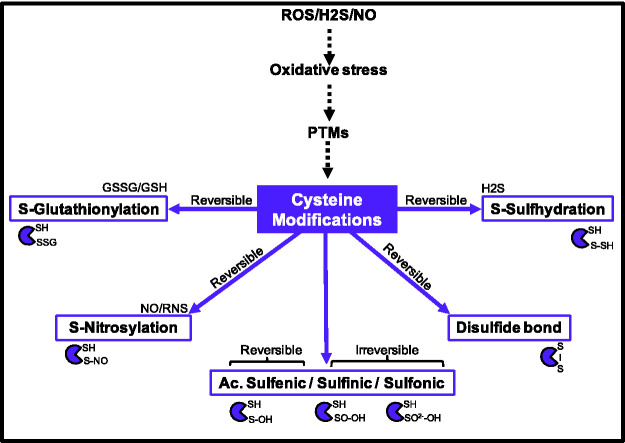
Overview of cysteine post-translational modifications (PTMs). GSH: Glutathione; GSSG: Glutathione disulfide; H_2_O_2_: Hydrogen peroxide; H_2_S: Hydrogen Sulfide; NO: Nitric Oxide; PTMs: post-translational modifications; RNS: reactive nitrogen species; ROS: reactive oxygen species.

Cysteines account for 2% of the total amino acid content of cells, which is the lowest number for all amino acids, but they are highly conserved and undergo oxidation and reduction (Miseta and Csutora, 2000). This suggests important biological roles for these amino acid residues such as redox-dependent modification and the complexing of metal ligands (Pace and Weerapana, 2013). Cysteine oxidation includes enzyme-mediated disulfide bond formation that generally enhances the structural activity and the folding of proteins (Lo Conte and Carroll, 2013). However, cysteines are also targets of oxidizing PTMs mediated by reactive chemicals (e.g., reactive oxygen species, ROS, reactive nitrogen species, RNS) (Chung et al., 2013), and by oxygen free radicals (Figure 1) (Takashima et al., 2012). Proteomic studies have listed the peptides undergoing such modifications, many of which found inside mitochondria and the ER (Finelli, 2020).

ROS and RNS-based cysteine modifications give rise to thiol-based redox regulation, whose importance competes with phosphorylation-based regulation. Cysteine oxidative modifications include reversible sulfenic acid (-SOH), sulfinic acid (-SO_2_H) and irreversible sulfonic acid (-SO_3_H). The partial reversibility of these oxidation-based PTMs highlights the labile aspect of these modifications, but also their role as on/off switches (Garcia-Santamarina et al., 2014). Preventing cysteine oxidation, peroxiredoxins scavenge ROS (Rhee, 2016). Once cysteines are oxidized, reduced forms of NADPH, glutathione, cysteine and thioredoxin can remove these PTMs (Miller et al., 2018). Particularly important for the maintenance of this function is the ratio between oxidized and reduced glutathione (GSSG, GSH), which is very responsive to changes in ROS (Mailloux, 2020). Glutathione can also act as a PTM itself via spontaneous or enzymatic modification of cysteines by ER-localized glutathione S-transferase (GST). Substrates of this enzyme include for instance ER chaperones like calnexin or immunoglobulin binding protein/glucose-regulated protein of 78 kDa (BiP/GRP78) (Ye et al., 2017; Scire et al., 2019). In contrast, glutaredoxins (GRX) 1 and 2 deglutathionylate these cysteine residues (Matsui et al., 2020).

Additionally, H_2_O_2_ can also react with nitric oxide (**^.^** NO) to yield peroxynitrite, a major RNS (Radi, 2018). RNS are also able to oxidize cysteines without the help of enzymes (Evangelista et al., 2013). Like ROS-based oxidation, these modifications can be reversible or irreversible and typically occur on cysteines with a charged residue in close proximity (Marino and Gladyshev, 2010). In its reversible form, S-nitrosylation is removed by enzymes, including S-glutathione reductase (Rizza and Filomeni, 2017).

Our review will first give an overview on the MERC proteome, followed by a list of MERC-associated cysteine oxidation-dependent PTMs and their functions for lipid and Ca^2+^ flux. We will also discuss potential cysteine PTM sources and the role of these modifications in disease.

## Tethering the Two Membranes of MERCs

**Figure 2.**
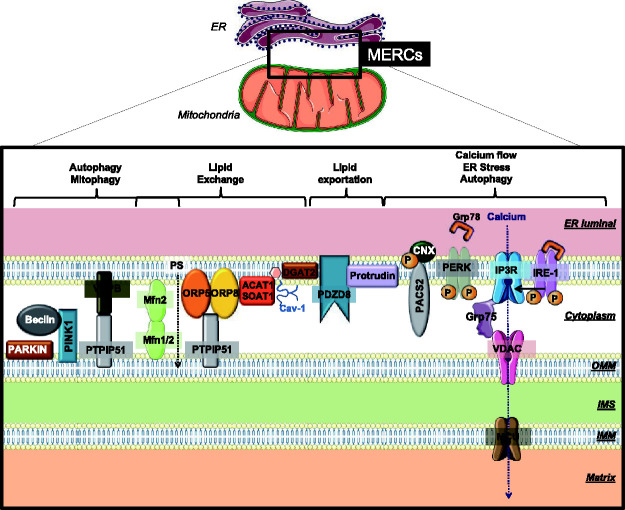
**Main protein complexes controlling mitochondria–ER contact sites (MERCs)**. Important tethers and carrier proteins are grouped according to their main function. BAP-31: B-cell receptor-associated protein of 31kDa; CNX: Calnexin; CypD: Cyclophilin D; ER: Endoplasmic Reticulum; Fis1: Mitochondrial Fission 1 Protein; Grp75: glucose-regulated protein of 75kDa (or mortalin/heat shock protein 75); Grp78: glucose-regulated protein of 78 kDa; IMM: inner mitochondrial membrane; IMS: inter-membrane space; IP3R: inositol 1,4,5-trisphosphate receptor; IRE-1: inositol-requiring enzyme 1; MCU: mitochondrial calcium uniporter; MERCs: mitochondria–ER contact sites; Mfn1/2: Mitofusin 1 and 2; OMM: Outer mitochondrial membrane; ORP5-8: oxysterol-binding protein-related protein 5 and 8; P: Phosphorylation; PACS2: Phosphofurin acidic cluster sorting protein 2; PERK: Protein kinase R-like ER kinase; PINK1: PTEN-induced kinase 1; PS: phosphatidylserine; PTPIP51:protein tyrosine phosphatase interacting protein 51; VAPB: vesicle-associated membrane protein-associated protein B; VDAC: voltage-dependent anion channel.

The apposition of the ER with mitochondria on MERCs depends on the assembly of protein tethers and their regulatory proteins, including PACS-2 that is required for ER-mitochondria apposition and Ca^2+^ flux (Simmen et al., 2005) (Figure 2). An example of an ER-mitochondria tethering complex is the interaction between the vesicle-associated membrane protein-associated protein B (VAPB) and protein tyrosine phosphatase interacting protein 51 (PTPIP51), located at the ER and at the outer mitochondrial membrane (OMM), respectively (Stoica et al., 2014; Gomez-Suaga et al., 2017). This complex also plays a role in autophagy. Its formation is disrupted upon activation of the redox-sensitive glycogen synthase kinase 3β (GSK3β) (Stoica et al., 2016).

In yeast, the ER-mitochondria encounter structure (ERMES) links ER and mitochondrial membranes, mediating a regulated lipid conduit (Kornmann et al., 2009). As is typical for tethering complexes, its individual components Mmm1, Mdm10, Mdm12 and Mdm34 localize to the ER and mitochondria, respectively and their assembly is controlled by the GTPase Gem1 (Kornmann et al., 2011). Whether this complex also exists in mammalian cells had long been discussed (Wideman et al., 2018). The discovery of the PDZ domain protein PDZD8, an Mmm1 paralog, as a mediator of ER-mitochondria and ER-late endosome-mitochondria contact sites suggests that aspects of this membrane tether are conserved in mammalian cells but its function could be more complex (Hirabayashi et al., 2017). At multi-organelle contact sites, PDZD8 also interacts with ER-localized protrudin and endosomal Rab7 (Elbaz-Alon et al., 2020) and as a synaptotagmin-like mitochondrial lipid-binding proteins (SMP), PDZD8 extracts lipids from the ER and transfers them to late endosomes (Shirane et al., 2020). Another PDZ domain is found within the mitochondrial synaptojanin-2 binding protein (Synj2BP) that has been discovered in a proteomic screen (Hung et al., 2017). Syn2BP forms a tether with the ER-localized ribosome binding protein 1 (RRBP1), but also interacts with the transmembrane and immunoglobulin domain containing protein 1 (TMIGD1) in epithelial cells (Hartmann et al., 2020). ER and mitochondria connections are also under the influence of actin polymerization. ER-bound inverted formin 2 (INF2) catalyzes actin polymerization that promotes the activity of mitochondrial Drp1 and the transfer of Ca^2+^ from the ER to mitochondria (Korobova et al., 2013; Chakrabarti et al., 2018).

The first characterized MERC protein that controls tethering is mitofusin-2, a dynamin-related GTPase. Mitofusin-2 can form proteinaceous bridges between the ER and mitochondria (de Brito and Scorrano, 2008), but this function could also determine the nature and extent of interactions between mitochondria and the ER (Filadi et al., 2015). Specifically, mitofusin-2 could control the ratio of rough to smooth ER contacts with mitochondria (Wang et al., 2015). In parallel with the MERC-regulatory roles of PACS-2, these interactions are essential for the induction of autophagy with MERCs as the source material (Hamasaki et al., 2013). Moreover, PACS-2 controls an ER-mitochondria protein complex called the ARCosome, which is composed of ER-localized BAP31 and mitochondrial Fis1 (Iwasawa et al., 2011). More tethers may be discovered in the future and the nature and function of these may differ between tissue sources, since multiple proteomic studies have identified different numbers and identities of proteins found within the biochemical MAM isolate (Cho et al., 2017; Hung et al., 2017).

## Redox Control of MERC Tethers and Mitochondrial Membrane Dynamics

**Figure 3.**
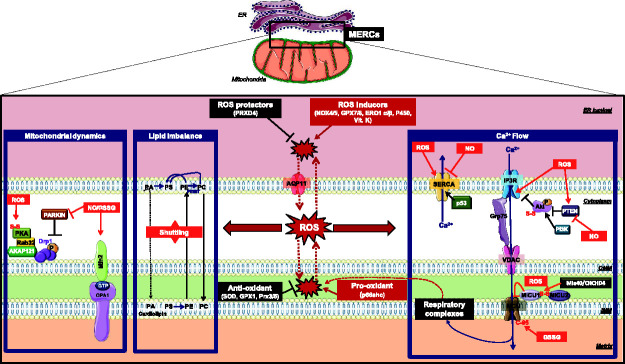
**ROS regulation and cysteine impact on mitochondria–ER contact sites protein players**. Important ROS-sensitive MERC proteins are grouped according to their main function, including mitochondrial dynamics, lipid transfer and Ca^2+^ flow. ROS sources and sinks are indicated. AKAP121: A kinase anchor protein of 121kDa; AQP11: aquaporin 11; Ca^2+^: Calcium; Drp1: Dynamin-related protein 1; ER: endoplasmic Reticulum; ERO1α/β: endoplasmic Reticulum oxidoreductin 1 α/β; GPX1: Glutathione peroxidase; GPX7/8: glutathione Peroxidase 7; Grp75: glucose-regulated protein of 75kDa (or mortalin/heat shock protein 75); GSSG: oxidized glutathione; H_2_O_2_: hydrogen peroxide; IMM: inner mitochondrial membrane; IMS: inter-membrane space; IP3R: inositol 1,4,5-trisphosphate receptor; MCU: mitochondrial calcium uniporter; MERCs: mitochondria–ER contact sites; Mfn2: mitofusin2; MICU1/2: mitochondrial Ca^2+^ uptake proteins 1 and 2; NO: nitric Oxide; NOX4/5: NADPH oxidase 4/5; OMM: outer mitochondrial membrane; OPA1:mitochondrial dynamin like GTPase; P53: dellular tumor antigen p53; P450: hemoprotein cytochrome P450; PI3K: Phosphoinositide 3-kinases; PKA: protein kinase A; PRDX4: peroxiredoxin-4; Prx3/5: Peroxiredoxin 3/5; PTEN: phosphatase and TENsin homolog; Rab32: Ras-related protein Rab-32; ROS: reactive oxygen species; SERCA: sarco/endoplasmic reticulum Ca2+-ATPase; SOD: superoxide dismutase.

The tethering of the ER to mitochondria increases upon oxidative stress and relaxes upon homeostatic conditions (Csordas et al., 2006). This oscillating interaction coincides with ER Ca^2+^ release that then activates mitochondrial oxidative phosphorylation (OXPHOS) associated with a burst of ROS entering the interorganellar cleft (Bravo et al., 2011; Booth et al., 2016). This suggests that some or all of the MERC tethers are under the control of redox PTMs. However, only a few PTMs of MERC tethers are currently known and most of our knowledge is limited to proteins with functions in mitochondrial membrane dynamics, including Drp1 (Friedman et al., 2011) and mitofusin-2 (de Brito and Scorrano, 2008) (Figure 3).

One example is mitofusin-2, which is subject to ROS-mediated PTMs that determine its role in mitochondrial membrane fusion (Shutt et al., 2012; Mattie et al., 2018). Under oxidizing conditions, an increase of GSSG concentration at MERCs leads to Mfn2 recruitment. Subsequently, glutathionylation of cysteine 684 cooperates with mitofusin-1 to promote mitochondrial fusion (Shutt et al., 2012). Interestingly, such Mfn1-Mfn1 dimers are more than a hundred times stronger than homodimers between Mfn2-Mfn2 (Ishihara et al., 2004). Cysteine PTMs could also impact the role of mitofusin-2 at MERCs, since MERC-originating autophagy accelerates in the presence of ROS (Forte et al., 2017). An additional level of mitofusin redox control derives from the ROS/RNS-mediated activation of c-Jun N-terminal kinases (JNK) (Kamata et al., 2005), which normally promote mitofusin-2 degradation by the proteasome (Leboucher et al., 2012). Therefore, oxidative conditions eventually reduce the amounts of mitofusins.

Opposing mitofusins, the OMM GTPase Drp1 uses receptors to associate with the mitochondrial membrane (Kamerkar et al., 2018) and constrict mitochondria (Friedman et al., 2011). To date, Fis-1 and mitochondrial fission factor (Mff) have been identified as further regulatory proteins whose regulation via oxidation is uncharacterized (Wolf et al., 2020). In contrast, mitochondrial dynamics proteins of 49 or 51 kDa (MID49/MID51) can undergo oligomerization under oxidizing conditions (Zhao et al., 2011). Oxidative stress activates mitochondrial fission in multiple ways. First, it directly recruits and activates Drp1 through S-nitrosylation on cysteine 644, which serves as a trigger for oligomerization (Cho et al., 2009) and further activation via phosphorylation of serine 616 (Lee and Kim, 2018). In the proximity of the ER, a moiety of protein disulfide isomerase (PDI) that localizes to the cytosol could directly inhibit this activity via catalytically reducing oxidized Drp1 (Kim et al., 2018). These activities are likely impaired in the presence of ROS and other mitochondrial compounds, since excess fumarate from mitochondria succinates and inactivates PDI (Manuel et al., 2017). Another connection between Drp1 and cellular redox conditions is mediated by small ubiquitin-like modifier (SUMO), a covalent modification of lysine residues that controls protein-protein interactions (Flotho and Melchior, 2013). In the case of Drp1, this modification is mediated by the MERC-associated mitochondrial-anchored RING finger containing protein (MAPL), a SUMO E3 ligase. Its activity SUMOylates and oligomerizes Drp1, thus increasing MERC formation and ER-mitochondria Ca^2+^ communication (Braschi et al., 2009; Prudent et al., 2015). SUMO isopeptidases called sentrin-specific proteases (SENPs) remove these modifications. However, they may undergo inactivation via an intramolecular disulfide bond in the presence of ROS (Ferdaoussi et al., 2015). Thus, while SENP5 normally destabilizes Drp1 (Zunino et al., 2009), this may not occur during oxidative stress, resulting in the accumulation of active Drp1. In contrast, SUMO E1 and E2 ligases can become inactivated by reversible oxidation of their catalytic cysteines in the presence of ROS (Bossis and Melchior, 2006). Thus, the interplay between ROS and SUMOylation is complex and warrants further investigation.

Another Drp1-activating mechanism is based on ubiquitination. Parkin, a regulatory protein of mitochondrial membrane dynamics that localizes to MERCs is a cytosolic E3 ubiquitin ligase (Gelmetti et al., 2017). This allows Parkin to target Drp1 for proteasomal degradation (Wang et al., 2011). However, upon its S-nitrosylation, Parkin no longer promotes the degradation of Drp1 (Chung et al., 2004; Gelmetti et al., 2017). Overall, it appears that oxidative stress activates Drp1 to promote mitochondrial fission. This role is particularly important in the central nervous system (CNS), where oxidative stress derived from neurodegeneration coincides with active Drp1 and inactive Parkin (Ge et al., 2020), resulting in compromised mitophagy and increased mitochondrial fragmentation (Cho et al., 2009).

MERCs also influence mitochondria movement along the cytoskeleton with the help of kinesin and dynein (Yi et al., 2004). These two motor proteins interact with the small mitochondrial Rho proteins 1 and 2 (Miro1/2), which are both enriched in MAMs. Their Ca^2+^-binding EF hand domains detach from kinesin in the presence of high Ca^2+^, arresting mitochondria movement (Fransson et al., 2006). Consistent with the role of ROS as a booster of Drp1-mediated MAM formation, mitochondrial ROS decrease Miro-mediated movement of mitochondria in a parallel mechanism that is independent of Ca^2+^ but requires the p38 MAP kinase (Debattisti et al., 2017).

## Redox Control of Mitochondria Metabolism Through MERC Ca^2+^ Transfer

In most cell types, the free mitochondrial Ca^2+^ concentration of 100 to 200 nM resembles the one found in the cytoplasm. These amounts are, however, about 1000 to 8000 times lower than what is observed in the ER (100 to 800 μM) and remain much lower than the typical extracellular medium that is usually situated at about 2 mM (Rizzuto et al., 2009). However, the mitochondrial free [Ca^2+^] content undergoes fluctuations dependent on ER Ca^2+^ release, for instance through the activation of IP_3_Rs with histamine, which raises mitochondrial free [Ca^2+^] into the low micromolar range, as summarized by (Fernandez-Sanz et al., 2019). Following its release from the ER, physical interactions between mitochondria and the ER allow for Ca^2+^ transfer into mitochondria (Rizzuto et al., 1998). Such an increase of mitochondrial [Ca^2+^] not only promotes the Krebs cycle but can also result in apoptosis (Jouaville et al., 1999; Pinton et al., 2001). The import and release of Ca^2+^ on the mitochondrial membranes is therefore critical for the control of cell fate and metabolism. Changing redox conditions within and around mitochondria control the functioning of mitochondrial Ca^2+^ gatekeepers. Of particular interest is the mitochondrial intermembrane space (IMS). This compartment contains large amounts of ROS-consuming or generating enzymes such as superoxide dismutase 1 (SOD1), peroxiredoxins (PRDX) and glutathione peroxidases (GPx) that are able to buffer ROS generated from mitochondrial OXPHOS (Riemer et al., 2015). These groups of enzymes also control the folding of proteins imported into mitochondria from the cytosol via the TOM and TIM complexes (Habich et al., 2019). Within the IMS, a disulfide relay system composed of the oxidoreductase CHCHD4 (known in yeast as Mia40) and the sulfhydryl oxidase ALR (known in yeast as Erv1) catalyzes the correct formation of disulfide bonds of mitochondrial IMS and IMM proteins (Fischer et al., 2013). Consistent with an important role of IMS redox conditions for their folding and functioning, many IMS proteins contain conserved cysteine residues, including mitochondrial Ca^2+^-handling proteins (Vogtle et al., 2012; Hung et al., 2014). Of particular interest is the redox-sensing protein p66Shc (Giorgio et al., 2005). p66Shc can induce mitochondrial ROS synthesis by sequestering cytochrome c from the respiratory chain (Giorgio et al., 2005) or by increasing the rate of OXPHOS (Lone et al., 2018). At the same time, p66Shc inhibits the expression of antioxidant enzymes such as SOD1 (Lebiedzinska et al., 2009). Under oxidizing conditions, p66Shc uses N-terminal cysteine residues to form a tetramer that promotes mitochondrial permeability transition (Gertz et al., 2008).

**Figure 4.**
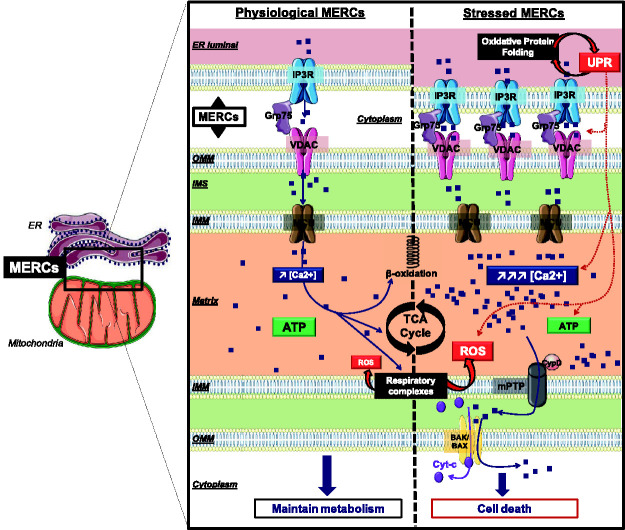
**ROS and stress-dependent Ca^2+^ signaling at mitochondria–ER contact sites**. Distinct ROS signaling patterns adapt the MERC structure to allow for changes in Ca^2+^ signaling and TCA cycle activity. ATP: Adenosine triphosphate; BAK/BAX: Bcl-2-associated X protein; Ca^2+^: Calcium; CypD: Cyclophilin D; Cyt-c: Cytrochrome-c; ER: Endoplasmic Reticulum; Grp75: glucose-regulated protein of 75kDa (or mortalin/heat shock protein 75); IMM: inner mitochondrial membrane; IMS: inter-membrane space; IP3R: inositol 1,4,5-trisphosphate receptor; MCU: mitochondrial calcium uniporter; MERCs: mitochondria–ER contact sites; mPTP: mitochondrial permeability transition pore; OMM: Outer mitochondrial membrane; ROS: reactive oxygen species; SERCA: sarco/endoplasmic reticulum Ca^2+^-ATPase; TCA cycle: tricarboxylic acid cycle; VDAC: voltage-dependent anion channel.

Ca^2+^ enters the IMS through the porin-related VDAC channels, of which VDAC1 is the predominant isoform (De Stefani et al., 2012; Messina et al., 2012). Thus, the OMM-localized VDAC1 can limit redox-regulated mitochondrial Ca^2+^ import (Figure 4) (Shoshan-Barmatz et al., 2018). VDAC1 contains two redox-responsive cysteine residues, but they do not affect its function, in contrast to VDAC2-3 isoforms (De Pinto et al., 2016). Rather, the redox-sensitive [2S-2Fe] cluster protein mitoNEET can provide a gating function to VDAC1 whose full significance for Ca^2+^ is currently unclear (Lipper et al., 2019).

From the IMS, the inner mitochondrial membrane (IMM)-localized mitochondrial Ca^2+^ uniporter (MCU) transfers Ca^2+^ into the matrix (Baughman et al., 2011; De Stefani et al., 2011). The MCU is a pentameric protein complex of transmembrane proteins with the N- and C-termini exposed to the mitochondrial matrix (Nemani et al., 2018). The MCU protein contains a cysteine residue in its matrix-localized N-terminal domain that undergoes glutathionylation in the presence of excess ROS from mitochondrial oxidative stress. Upon this modification, MCU undergoes higher order oligomerization, which increases mitochondrial Ca^2+^ entry, eventually resulting in mitochondrial Ca^2+^ overload (Dong et al., 2017). The MCU is controlled by mitochondrial Ca^2+^ uptake proteins 1 and 2 (MICU1 and MICU2) (Patron et al., 2014), and the essential MCU regulator (EMRE) which together form a 480 kDa complex anchored in the IMM (Sancak et al., 2013). We name this assembly the “MCU complex” henceforth. The MCU complex is inhibited by MICU2 in low Ca^2+^ concentrations, but in high Ca^2+^ concentrations, MICU1 stimulates the MCU complex and allows Ca^2+^ entry into the mitochondrial matrix (Nemani et al., 2018). The oxidoreductase Mia40/CHCHD4 catalyzes the formation of a disulfide bond between MICU1 and MICU2, which attenuates mitochondrial Ca^2+^ entry (Petrungaro et al., 2015). Therefore, cysteine oxidation of the MCU channel in the matrix acts to overload mitochondria with Ca^2+^, but Mia40/CHCHD4 opposes this activation within the IMS. Although the MCU complex is not particularly enriched on MERCs, the majority of the Ca^2+^ it receives originates from the ER (Qi et al., 2015). Increased or decreased amounts of the oxidoreductase Ero1α could influence MCU Ca^2+^ uptake, but it is currently not known how this affects the components of the MCU complex (Anelli et al., 2011). Regardless of this uncertainty, redox signaling can radiate out from one organelle to the other, as seen for example during ER stress, which causes an increase of the mitochondrial Lon protease that degrades oxidized mitochondrial proteins (Hori et al., 2002). Moreover, the transmission of mitochondrial ROS can induce the ER unfolded protein response (UPR) (Yoon et al., 2011).

## Control of MERC Ca^2+^ Signaling by Cysteine PTMs on the ER

The extent of ER Ca^2+^ content determines the extent of Ca^2+^ flux towards mitochondria (Gutierrez et al., 2020). Store operated Ca^2+^ entry (SOCE) (Elaib et al., 2016) and Ca^2+^ import by sarco/endoplasmic reticulum Ca^2+^-ATPase (SERCA) pumps combine to store Ca^2+^ within the ER (Primeau et al., 2018). Although full-length SERCA shows no enrichment to any ER domain (Raturi et al., 2016), truncated forms of SERCA can localize to MERCs (Chami et al., 2008). Like most MERC Ca^2+^-handling proteins (Figure 3), SERCA is subject to extensive redox regulation (Chernorudskiy and Zito, 2017). SERCA activity can increase under oxidative conditions (Adachi et al., 2004) and this activity determines MERC functioning (Raturi et al., 2016). ER-localized NADPH oxidase 4 (Nox4) mediates this baseline activation (Evangelista et al., 2012). While the ER chaperone calnexin maintains Nox4 activity (Prior et al., 2016) and, hence, SERCA pumping (Gutierrez et al., 2020), the reductase TMX1 inhibits SERCA and prevents the re-capture of the cytosolic Ca^2+^ (Figure 4) (Raturi et al., 2016). Overall, a baseline oxidation of SERCA correlates with full activity.

However, conflicting results have been reported on the overall level of SERCA cysteine oxidation, also regarding luminal or cytosolic cysteines, leading to a dichotomy of the SERCA redox regulation (Raturi et al., 2014). Accordingly, unlike the activating calnexin that increases SERCA oxidation, a number of ER proteins including ERdj5 and selenoprotein N (SELENON) activate SERCA pumps by reducing critical luminal cysteines (Marino et al., 2015; Ushioda et al., 2016). This is also seen for p53, which reduces the cytoplasmic oxidation of SERCA (Giorgi et al., 2015). Together, the extent of SERCA oxidation could lead to a bell-shaped activity curve (Raturi et al., 2014). Consistent with this hypothesis, SERCA activity decreases in the presence of hyper-oxidizing conditions, as found in aging tissue (Babusikova et al., 2012).

Once filled, the ER Ca^2+^ content can be released via ryanodine receptors (RyRs) or IP_3_Rs (Raffaello et al., 2016). All IP_3_R Ca^2+^ channels are enriched at MERCs but IP_3_R2 is most potent in transmitting Ca^2+^ from the ER to mitochondria (Bartok et al., 2019). Similarly, RyRs localize to MERCs (Chen et al., 2012), where they can generate an ER-mitochondria Ca^2+^ conduit in their own right (Eisner et al., 2013). Both IP_3_Rs (Beretta et al., 2020) and RyRs (Sun et al., 2011) are associated with Nox4, suggesting this ER ROS source fulfills a central role in the control of ER-mitochondria Ca^2+^ flux, apparently to maintain physiological ATP production (Eisner et al., 2013).

RyR1 oxidation and glutathionylation increases Ca^2+^ release associated with the redox-dependent dissociation from its regulatory proteins FKBP12 and calmodulin (Aracena et al., 2005; Aracena-Parks et al., 2006). IP_3_Rs act directly on MERCs, since they form a physical link with OMM-localized VDAC1 under the control of the mitochondrial chaperone Grp75 to generate a Ca^2+^ conduit toward mitochondria. This complex is also the first description of a MERC tethering complex (Szabadkai et al., 2006). Cytosolic H_2_O_2_ molecules lead to oxidation of two cysteine residues within the cytosolic suppressor domain of IP_3_R1 (cysteines 206, and 214) and one additional cytosolic residue (cysteine 1394) in addition to 5 cysteines that are already oxidized under basal conditions (Joseph et al., 2018). These modifications of the sulfenylation and sulfinylation type activate IP_3_R1. Therefore, oxidative stress increases Ca^2+^ flux through IP_3_Rs and may lead to a feed forward ER Ca^2+^ flux towards mitochondria. As a further consequence, this Ca^2+^-based feed forward mechanism triggers the release of H_2_O_2_ from mitochondria into the MERC cleft that increases ER-mitochondria tethering (Booth et al., 2016) (Figures 3 and 4).

Another mechanistic connection between ER Ca^2+^ channel activity and redox depends on ER chaperones. One example is the Sigma 1 receptor (SIGMAR1), an ER chaperone with a limited number of substrates (Hayashi and Su, 2007). Normally, SIGMAR1 is complexed to the ER chaperone BiP/GRP78. Upon detachment from BiP/GRP78 during ER stress, SIGMAR1 interacts with and activates IP_3_R, thus increasing Ca^2+^ transfer from the ER to mitochondria (Hayashi and Su, 2007). In parallel, ERp44 and Ero1α, two proteins controlling the ER folding and redox environment, inhibit IP_3_Rs under reducing conditions and activate it under oxidizing conditions (Higo et al., 2005; Li et al., 2009). ER stress is a condition that activates the formation of MERCs and likely causes a feed-forward mechanism analogous to the one originating at mitochondria (Booth et al., 2016), but based on dysfunctional ER oxidative protein folding (Csordas et al., 2006; Bravo et al., 2011) (Figure 4). This leads to a mechanistic connection between ER oxidative protein folding and ER-mitochondria Ca^2+^ flux (Simmen et al., 2010; Fan and Simmen, 2019) that is associated with increased passive ER Ca^2+^ leak during ER stress (Hammadi et al., 2013) and interactions of ER folding enzymes with SERCA pumps (John et al., 1998; Li and Camacho, 2004; Lynes et al., 2013).

MERC regulation by redox likely extends to the core of the UPR machinery, including protein kinase RNA-like endoplasmic reticulum kinase (PERK) (Harding et al., 1999; Hori et al., 2002). Consistent with a central role in MERC formation, the deletion of PERK reduces ER-mitochondria contact points associated with resistance to apoptosis (Verfaillie et al., 2012). Another major ER stress sensor is the inositol-requiring enzyme 1 (Ire1). A portion of Ire1 localizes to MERCs, where it acts as a scaffold for IP_3_Rs to control mitochondria Ca^2+^ transfer and metabolism (Carreras-Sureda et al., 2019). PERK and Ire1 may functionally link oxidative stress to MERC signaling via their UPR signaling properties that increase upon ROS incubation for PERK (Higa and Chevet, 2012) and Ire1 (Hourihan et al., 2016). However, how exactly these transmembrane ER stress sensors manipulate ROS signaling on MERCs remains to be investigated.

## MERC Lipid Homeostasis Is Linked to Ca^2+^ Flux and ER Stress and OXPHOS

The originally discovered function of MERCs is the production of specific lipids, as unveiled by the biochemical isolation of MAMs by Jean Vance (Vance, 1990, 1991). Indeed, the mitochondria ER membrane contact site (MCS) is a major hub in lipid biosynthesis (Figure 2) (Vance, 2015). MERCs have raft-like properties and form membrane microdomains enriched in sphingolipids and cholesterol (Sano et al., 2009; Hayashi and Fujimoto, 2010). This leads to the localization of Acetyl-Coenzyme A acetyltransferase 1 (ACAT1), also known as acyl-Coenzyme A: cholesterol acyltransferase 1 (SOAT1) to MERCs (Lee et al., 2000) where this enzyme esterifies and detoxifies cholesterol (Rogers et al., 2015). Once synthesized, cholesterol transfers over to mitochondria dependent on caveolin-1 that promotes MAMs and decreases free cholesterol (Sala-Vila et al., 2016).

Upon establishment of their raft-like structure, MERCs also supply mitochondrial phosphatidylcholine (PC), phosphatidylinositol (PI), and phosphatidylserine (PS) (van Meer et al., 2008). After PS production in the ER from PA (phosphatidic acid) and its transfer to mitochondria, PS is enzymatically converted to phosphatidylethanolamine (PE), one of two biosynthetic pathways for this lipid (Schuiki and Daum, 2009). Mitochondrial PE is cycled back to the ER, where it is transformed into PC by the action of phosphatidylethanolamine N-methyltransferase (PEMT) (Vance, 2015).

Imbalance of these enzymatic reactions induces ER stress and subsequently triggers MERC dysfunction, highlighting the symbiotic relationship between the ER and mitochondria. Consistent with this, compromised MERC lipid homeostasis, for instance from disrupted PS shuttling usually leads to ER stress (Hernandez-Alvarez et al., 2019). UPR induction also results from increased palmitate loading of the ER (Karaskov et al., 2006), a lack of PEMT (Gao et al., 2015) or from reduced PC levels (Hou et al., 2014). Mechanistically, these conditions of disrupted lipid homeostasis typically trigger mitochondrial ROS production (Xu et al., 2015), but can also impair mitochondrial ATP production, mitochondrial morphology and assembly of OXPHOS components (Tasseva et al., 2013). There is therefore a functional nexus between proteins controlling the MERC lipidome, the UPR and mitochondrial functions. Another example is the interorganellar PS/PE shuttling, which requires oxysterol-binding proteins 5 and 8 (ORP5/8) (Chung et al., 2015). This lipid shuttle also operates on MERCs, where ORP5/8 interact with PTPIP51 on the OMM (Galmes et al., 2016). Through their function for the PC/PE ratio, ORP5/8 allow for normal respiration. If over-expressed they improve mitochondrial Ca^2+^ import (Pulli et al., 2018). As expected, altering the function of this lipid shuttle, for instance via over-expression of ORP8, induces ER stress (Guo et al., 2017).

At the root of these observations may be the depletion of the ER Ca^2+^ content that is transferred over to mitochondria. This could occur for instance upon an elevation of the PC/PE ratio, which inhibits SERCA pumps by decreasing their Ca^2+^ affinity (Gustavsson et al., 2011). Thus, the altered MERC lipidome may disrupt proper Ca^2+^ filling of the ER, which is not only critical for ER protein folding (Simmen and Herrera-Cruz, 2018), but also for mitochondrial oxidative phosphorylation (Gutierrez et al., 2020). Another functional link is provided by the shuttling of ER-synthesized phosphatidic acid (PA) towards mitochondria to be enzymatically transformed into cardiolipin, which is essential for mitochondrial structure and function (Osman et al., 2011; Potting et al., 2013). Like reduced PE levels, interference with cardiolipin synthesis leads to ER stress, culminating in the activation of the C/EBP homologous protein (CHOP) (Sustarsic et al., 2018).

Lipids themselves are sensitive to ROS. An increase of lipid peroxidation compromises the folding environment of the ER and can lead to a feed forward mechanism of progressing dysfunction (Lin et al., 2014). Thus, MERCs become dysfunctional under extended oxidizing conditions (Janikiewicz et al., 2018). Ultimately, lipid peroxidation impairs mitochondrial OXPHOS (Anderson et al., 2012). Cardiolipin is also susceptible to peroxidation, which severely compromises mitochondrial OXPHOS (Paradies et al., 2009). Upon Bax/Bak-mediated OMM pore formation during apoptosis, cytochrome c can induce cardiolipin peroxidation following the formation of a complex with cardiolipin peroxidase, which then accelerates cell death (Kagan et al., 2005). Less is known about links between the IMS folding environment and lipid homeostasis, but the generation of oxidized sterols leads to the recruitment of the ubiquitin proteasome system to remove the mitochondrial OMM protein import machinery in yeast, suggesting this compartment could be affected in similar ways to the ER folding environment (Nielson et al., 2017).

The importance of MERC redox changes extends to lipid-related downstream effects. The biosynthesis of triacylglycerol (TG) is under the control of two diacyglycerol acyltransferases (DGAT1/2), of which DGAT2 is MERC enriched (Stone et al., 2009). The activity of both enzymes is arrested upon incubation with thiol-modifying compounds (Sauro and Strickland, 1990), because the oxidation of cysteines blocks DGATs (Jung et al., 2017).

## Sources of Cysteine Post-Translational Modifications

MERCs are a convergence point for ROS produced within the ER, mitochondria and peroxisome (Yoboue et al., 2018). ROS production increases upon the arrival of growth factor and cytokine signaling (Nam et al., 2010). Within the ER, ROS are made from oxidative protein folding that requires oxygen consumption by the oxidoreductases of the Ero1 family (Araki et al., 2013), as well as by other enzymes such as the hemoproteins cytochrome P450 (Guengerich, 2019), and Nox4/5 (Laurindo et al., 2014). The Ero1 flavoproteins act together with glutathione peroxidases (GPx) 7/8 and PRDX4 to generate oxidized protein disulfide isomerase (PDI) (Bulleid and Ellgaard, 2011). Ero1 exists in humans as the hypoxia-controlled Ero1α (May et al., 2005) and the ER-stress regulated Ero1β (Cabibbo et al., 2000; Pagani et al., 2000). Both GPx7 and GPx8 act as peroxidases to promote the oxidation of substrate disulfide bonds in the presence of PDI (Nguyen et al., 2011). GPx7 is a luminal protein, while GPx8 spans the ER membrane (Nguyen et al., 2011; Kanemura et al., 2020). PRDX4 assists Ero1 oxidoreductases to eliminate excess H_2_O_2_ produced from oxygen consumption and uses it for PDI oxidation (Rhee et al., 2018). While Ero1α and GPx8 are known MERC proteins, we currently do not know the intra-ER localization of the other ER-based ROS sources and sinks (Gilady et al., 2010; Yoboue et al., 2018).

The ER-localized oxidoreductive network based on Ero1, GPx7/8 and PRDX4 is intimately linked with the redox state of the cellular volume adjacent to the ER, which potentially includes mitochondria. Accordingly, the regeneration of NADPH within the cytosol is critical for PDI oxidation (Poet et al., 2017) and the same is true for cytosolic thioredoxin reductase (Cao et al., 2020). These observations must be integrated with the inability of ROS to diffuse freely within the cell (Appenzeller-Herzog et al., 2016). Aquaporin-11 and other aquaporin family members have been identified as mediating ROS transport across the ER and other membranes, suggesting these proteins could be critical for MERC ROS communication (Bestetti et al., 2020).

Peroxisomes and mitochondria are the alternative MERC-relevant ROS producers. Within the mitochondria, complex I and III of the electron transport chain can produce ROS if there is a backup of electron flow (Murphy, 2009). Alternative sources are activated upon high levels of matrix NADH/NAD+ that are a consequence of reverse electron flow (Robb et al., 2018). Additionally, β-oxidation of fatty acids also promotes mitochondrial ROS production in some tissues, but notably not in the brain (Schonfeld and Reiser, 2017). Large quantities of ROS are stored within the mitochondrial cristae, which can be released upon increased Ca^2+^ influx from the ER, inducing a feed forward mechanism (Booth et al., 2016). To a currently unknown extent, antioxidant defenses found within the IMS like SOD1 (Kawamata and Manfredi, 2010), GPx and PRDXs (Mailloux et al., 2013) could potentially absorb them (Dimayuga et al., 2007).

Within peroxisomes, the oxidation of very long chain fatty acids and amino acids uses the enzymatic activity of flavoproteins, whose ROS production is scavenged by catalase and PRDX5 (Bonekamp et al., 2009). The influence of this ROS moiety on the ER and mitochondria is currently under debate (Lismont et al., 2015; Yoboue et al., 2018). Further research will show how these three sources are integrated and controlled under physiological and stress conditions.

## Metabolic and Apoptotic Signaling as a MERC-Localized PTM Target

The physiological influx of Ca^2+^ from the ER to mitochondria activates mitochondrial dehydrogenases, including glycerol-3-phosphate dehydrogenase (GPDH), pyruvate dehydrogenase (PDH), isocitrate dehydrogenase (IDH) and oxoglutarate dehydrogenase (OGDH) (Denton, 2009). ATP synthase (Jouaville et al., 1999) and β-oxidation (Balu et al., 2016) also appear to be a target of Ca^2+^ regulation. Opposing the function of Ca^2+^, glutathionylation inhibits the Krebs cycle and OXPHOS (Kuksal et al., 2017). Upon a ROS-mediated oxidation of MERC Ca^2+^-handling proteins, the alteration of ER-mitochondria Ca^2+^ flux could further boost ROS production within mitochondria (Brookes et al., 2004). Such a metabolic shift could result in reduced glycolysis, associated with glutathionylation of glyceraldehyde-3-phosphate dehydrogenase (GAPDH) that decreases glycolytic flux, only reversed by increased NADPH or antioxidants (Mullarky and Cantley, 2015).

In contrast, excessive mitochondrial Ca^2+^ uptake leads to an overload, which further increases the production of ROS by the respiratory chain, especially if associated with the depolarization of the IMM and the opening of the mitochondrial permeability transition pore (Adam-Vizi and Starkov, 2010). Such an event coincides with the oligomerization of pro-apoptotic Bcl2 family proteins Bax and Bak into a pore structure (Flores-Romero et al., 2020). Subsequently, Ca^2+^ and cytochrome c are released from this pore and apoptosis ensues (Salvador-Gallego et al., 2016; Cosentino and Garcia-Saez, 2017). Therefore, under conditions when the cyclic ER-mitochondria Ca^2+^ flux exceeds the normal mitochondrial buffering capacity, the ER can also contribute to the triggering of cell death, using Ca^2+^ as a messenger (Pinton et al., 2008).

Another example how MERCs mechanistically connect ROS and Ca^2+^ signaling is based on uncoupling protein 3 (UCP3), which reduces ATP production and thus compromises SERCA pumping on the ER side of MERCs, highlighting additional connections between the ER and mitochondrial ATP (De Marchi et al., 2011). This activity could increase in the presence of oxidative stress that activates UCP3 (Mailloux et al., 2011).

Multiple MERC-localized kinase-based signaling mechanisms could be subject to ROS modulation. For instance, the anti-apoptotic kinase Akt decreases MERC formation upon growth factor signaling (Betz et al., 2013). However, upon induction of oxidative stress, Akt forms an intramolecular disulfide bond, which results in its degradation, thus presumably boosting MERC Ca^2+^ signaling (Murata et al., 2003). This MERC activation depends on MERC-localized the phosphatase tensin homolog (PTEN) (Bononi et al., 2013). In contrast, H_2_O_2_-induced oxidation inactivates PTEN, thus increasing the anti-apoptotic, Akt-mediated MERC disruption, followed by proliferation (Kwak et al., 2010; Numajiri et al., 2011; Zhang et al., 2020).

An intriguing link between MERCs and redox signaling exists within the control of the circadian cycle. This mechanism allows cells and tissues to maintain homeostasis in a time-dependently changing environment. During the circadian cycle, the brain and muscle arylhydrocarbon receptor nuclear translocator protein 1 (BMAL1) regulates ROS production by controlling the activation of the antioxidant response transcription factor nuclear factor erythroid 2-related factor 2 (Nrf2) (Wible et al., 2018). Since Nox4 is one of the targets of BMAL1 (Anea et al., 2013), BMAL1 could modulate MERC signaling to control mitochondria metabolism (Alexander et al., 2020). This mechanism could involve the formation of a circadian cycle of Ca^2+^ oscillations (Ikeda and Ikeda, 2014).

## Cysteine Oxidation at MERCs and Disease

Given the many known mechanisms that compromise or alter the functioning of the ER and mitochondria, as well as cellular metabolism based on cysteine oxidation at MERCs, it is not surprising that this mechanism malfunctions in many metabolic disease settings.

### Examples of MERC Cysteine Modification in Neurodegenerative Diseases

Most neurodegenerative diseases involve oxidative stress (Chen et al., 2012). For instance, the S-nitrosylation of PTEN, Drp1, and Parkin is associated with Alzheimer’s and Parkinson’s disease, which would result in mitochondrial fragmentation and MERC dysfunction (Nakamura et al., 2013). Additional MERC-associated cysteine modifications are known to occur in these neurodegenerative diseases. For instance, in a murine model, Akt sulfhydration causes a worsening of Alzheimer’s disease elicited by a high level of Tau protein phosphorylation in the brain (Sen et al., 2020). In addition, the PP2Ac protein, an inhibitor of Akt on MAMs, shows less activity in Alzheimer’s disease upon introduction of thiol disulfide bonds in its catalytic subunit (Foley et al., 2007). Overall, such changes would result in increased formation of MERCs and increased transfer of Ca^2+^, lipids and sterols to mitochondria, which is indeed found in patient tissue, but it remains to be determined whether cysteine PTMs of MERC proteins are behind these observations (Hedskog et al., 2013; Montesinos et al., 2020). Changes in parkin cysteine PTMs are generally thought to compromise its activity and act to promote disease progression (Barodia et al., 2017). In contrast, the H_2_S-mediated Parkin sulfhydration may act to re-activate its enzymatic activity and, hence, to slow down PD progression (Vandiver et al., 2013).

Other neurodegenerative syndromes such as Huntington’s disease (HD) or amyotrophic lateral sclerosis (ALS) also show increased oxidative stress. Relevant for MERC regulation, a clear increase of nitrosylated PDI was observed in ALS (Chen et al., 2013). Within HD tissue and primary cells from mouse HD models, altered, dysfunctional MERCs are associated with fragmented mitochondria and oxidative stress (Cherubini et al., 2020).

### Examples of MERC Cysteine Modification in Cardiovascular Diseases

Cardiovascular diseases are highly correlated with increased levels of ROS (Peoples et al., 2019). A well-characterized effect of these is the Nox4-mediated oxidation of SERCA that promotes endothelial migration (Evangelista et al., 2012) and normal angiogenesis after ischemia (Craige et al., 2011). The correlation between Ca^2+^ fluxes, redox status and cysteine modifications is strong in cardiovascular diseases, especially upon myocardial ischemia and reperfusion. Under this condition, myocardial cells frequently undergo cell death due to an excessive flux of Ca^2+^ from the sarcoplasmic reticulum (SR)/ER to the mitochondria, causing mitochondrial Ca^2+^ overload and cell death that eventually triggers heart failure (Santulli et al., 2015). The MCU complex undergoes oxidation within the matrix-exposed N-terminal domain, but this does not occur dependent on MERC-derived ROS (Dong et al., 2017). 

### Examples of MERC Cysteine Modification on Metabolic Diseases

In the case of insulin resistance in diabetes, increased mitochondrial ROS levels are a potential causative mechanism (Anderson et al., 2009). A known consequence of diabetes-associated ROS induction is the inactivation of the SENP1 SUMO isopeptidase that normally promotes insulin exocytosis (Ferdaoussi et al., 2015) but also mitochondrial metabolic activity during fasting (Wang et al., 2019). Several proteins directly found at MERCs like Akt (Betz et al., 2013) and PTEN (Bononi et al., 2013) are dysfunctional in obesity and type 2 diabetic situations. For instance, a high concentration of NO inhibits Akt causing insulin resistance, while a low concentration of NO opposes this disease-promoting effect through PTEN S-nitrosylation (Numajiri et al., 2011). Moreover, redox events at MERCs in diabetes are associated with Drp1 sulfenylation and excessive fission of the mitochondrial network in endothelial cells. The ensuing mitochondrial fragmentation further increases ROS production and endothelial senescence and thus worsens the disease (Yu et al., 2006; Peng et al., 2012). Subsequently, these MERC defects increase inflammation whose hallmark is the activation of the inflammasome in the proximity of the ER and mitochondria (Zhou et al., 2011). Under this condition, interleukins and TNFa are secreted, which are two major components of inflammation following the activation of NFkB (Liu et al., 2017).

### Examples of MERC Cysteine Modification in Cancer

A plethora of cancer-relevant proteins controls metabolic MERC signaling, including p53 (Giorgi et al., 2015), PTEN (Bononi et al., 2013), the kinase Akt (Betz et al., 2013), breast/ovarian cancer susceptibility gene 1 (BRCA1) (Hedgepeth et al., 2015) and the promyelocytic leukemia (PML) protein (Giorgi et al., 2010; Missiroli et al., 2016). PML is very sensitive to oxidation (Tessier et al., 2017). While its MERC activity increases IP_3_R-mediated Ca^2+^ flux towards mitochondria, in its absence ROS increase (Niwa-Kawakita et al., 2017). Similarly, the transcriptional roles of p53 are highly redox-dependent (Kim et al., 2011). At MERCs, p53 reduces the cytosolic oxidation of SERCA and thus makes SERCA more active (Giorgi et al., 2015). Several additional redox-sensitive MERC proteins are recognized as oncogenic proteins, including proteins mediating the GSK3β pathways and frequently undergo oxidation in a cancer context (Koundouros and Poulogiannis, 2018; Zhang et al., 2020). While there is some evidence that MERCs are disrupted in a cancer context, thus promoting a Warburg metabolic signature with increased glycolysis (Herrera-Cruz and Simmen, 2017), MERC formation can also act as cancer-promoting. For example, the activity of the MCU complex promotes migration and invasion of breast cancer cells (Tosatto et al., 2016). Similarly, the activity of IP_3_Rs is necessary to prevent energy depletion of cancer cells (Cardenas et al., 2010). High expression of ROS-generating Ero1α worsens prognosis of breast cancer patients, which given its activating role for IP_3_Rs again suggests a cancer-promoting role (Kutomi et al., 2013). However, contradicting these findings, reduced expression of the BRCA1-associated protein 1 (BAP1) increases the incidence of cancer in parallel to a reduction of IP_3_R activity (Bononi et al., 2017). The complexity of redox-control of MERCs in cancer is possibly best illustrated by the reductase TMX1 that if depleted from melanoma cells can slow down their growth and mitochondria metabolism (Raturi et al., 2016), but in patients is often highly expressed to promote mitochondrial activity (Zhang et al., 2019). In summary, MERC disruption can arrest mitochondria metabolism in cancer, but the maintenance of mitochondria metabolism by functional MERCs can act as tumor-promoting by promoting invasion and metastasis (Denisenko et al., 2019).

To conclude, MERCs are a central cellular hub in metabolic and aging-related diseases. Redox control of MERC tethering and regulatory proteins is a key mechanism to control their roles in metabolism. Much of the information gathered on cysteine PTMs controlling MERCs has been gathered before their significance for this organellar contact site was known, suggesting that future research may reveal additional functional connections.
